# Infradiaphragmatic Hodgkin lymphoma: a large series of patients staged with PET-CT

**DOI:** 10.18632/oncotarget.19389

**Published:** 2017-07-19

**Authors:** Cédric Rossi, Morgane Mounier, Pauline Brice, Violaine Safar, Emmanuelle Nicolas-Virelizier, Philippe Rey, Aspasia Stamatoullas-Bastard, Marion Alcantara, Adrien Chauchet, Emilie Reboursière, Lauriane Filliatre, Aurore Perrot, Sylvain Garciaz, Gilles Salles, Bertrand Coiffier, Hervé Ghesquières, René-Olivier Casasnovas

**Affiliations:** ^1^ Hématologie Clinique, CHU Le Bocage, Dijon, France; ^2^ INSERM UMR 1037 - Cancer Research Center of Toulouse, Toulouse, France; ^3^ Registre des Hémopathies Malignes de Côte d’Or, EA4184, Université de Bourgogne, Dijon, France; ^4^ Hématologie Clinique, CHU Paris-GH St-Louis Lariboisière F-Widal - Hôpital Saint-Louis, Paris, France; ^5^ Hématologie Clinique, Centre Hospitalier Lyon Sud, Pierre-Bénite, France; ^6^ Hématologie Clinique, Centre Régional de Lutte Contre le Cancer Léon Bérard, Lyon, France; ^7^ Hématologie Clinique, Centre Henri Becquerel, Rouen, France; ^8^ Hématologie Clinique, CHU Besançon, Besançon, France; ^9^ Hématologie Clinique, CHU Caen, Caen, France; ^10^ Hématologie Clinique, CHRU Nancy, Nancy, France; ^11^ Hématologie Clinique, Institut Paoli-Calmettes, Marseille, France; ^12^ INSERM UMR 1231, Université Bourgogne Franche-Comté, Dijon, France

**Keywords:** Hodgkin lymphoma, infradiaphragmatic, radiotherapy

## Abstract

**Introduction:**

Infradiaphragmatic Hodgkin Lymphoma (IDHL) accounts for 3-11% of adult cases of stage I-II Hodgkin Lymphoma and the treatment strategy in IDHL is still heterogeneous. All previous published studies were conducted before the PET-CT era. PET may provide a more accurate evaluation of IDHL stage. The aim of this study was to analyze the clinical and biological characteristics of IDHL patients staged by CT scan or PET-CT in eight French hematology departments and their impact on outcomes in these patients.

**Methods:**

Baseline clinical and biological data and outcomes in patients with a first diagnosis of stage I-II IDHL treated with ABVD +/- radiotherapy were retrospectively collected.

**Results:**

Among the 99 patients included, 65 (66%) were staged with PET-CT. These patients were older (53 years vs 46 years, p=0.043), had lower ESR (27 vs 58mm, p=0.022), higher hemoglobin level (13.6 vs 12.8g/dL, p=0.015), less frequent Ann Arbor stage II (74% vs 91%) and less central adenopathy involvement (60% vs 82%, p=0.024). Treatment was chemotherapy alone in 55% of patients and the remaining patients received chemo-radiotherapy (CRT). Five-year PFS and OS rates in PET-CT-staged patients were 78% (95% CI 64-87) and 88% (95% CI 73-95), respectively, compared with 65% (p=0.225) and 82% (p=0.352) in CT-staged patients. The CRT strategy was associated with fewer relapses (p=0.027).

**Conclusion:**

This study showed that the characteristics of CT-staged IDHL patients were less favorable than those of PET-CT-staged patients and indicated that CRT provided better PFS than did chemotherapy alone.

## INTRODUCTION

Infradiaphragmatic Hodgkin lymphoma (IDHL) is an uncommon clinical entity of classical Hodgkin Lymphoma (HL), and accounts for 3-11% of adult cases of stage I-II HL [1-6]. Several specific characteristics of IDHL compared with supradiaphragmatic HL have been reported. These include the older age of patients, the predominance of males and more frequent mixed cellularity histology.

While the treatment strategy has been improved and standardized in recent decades in the main clinical subsets of HL, including supradiaphragmatic HL and advanced HL, the best treatment in IDHL is still a matter of debate. As these patients are often excluded from clinical trials and few cases of IDHL are reported in most retrospective published series, no consensual standard treatment or prognostic factors have emerged for the management of these patients. In addition, most series of IDHL [2-11] were published when only CT-scan was available for the staging and patients were often treated with radiotherapy alone [1, 3, 6-12]. PET-CT has better sensitivity and is more accurate than CT for identifying HL localization, in particular extra-nodal involvement, and has been recommended for staging HL patients since 2007 (Cheson 2007) [13-18].

We therefore performed a retrospective study in eight French institutions to compare the characteristics and outcomes of PET-CT-staged IDHL patients with those of CT-staged IDHL patients.

## RESULTS

### Patients’ characteristics at baseline

From 1986 to 2014, 99 untreated adults from eight French institutions were included. Among these, 89% (n=88) were diagnosed after 2000 and 45% (n=45) after 2004, date when PET-CT became available. The clinical and biological features of this cohort are reported in Table 1. Patients were predominantly male and almost half of them were over 50 years old. The disease was more often Ann Arbor stage II (80%), with central adenopathy (68%) and adenopathy localized in the lumbar-aortic (44%) and/or inguinal (75%) area. Most patients (77%) had a favorable performance status (PS=0). The ESR was frequently high with a median of 42.5 mm/h. The most common histological subtypes were nodular sclerosis (66%) and mixed cellularity (23%). The EBV status, assessed using EBERs analysis from tumor biopsies, was available in 59 patients and positive in 23 patients (39%). Ten percent of patients had bulky tumor masses (>10cm).

**Table 1:** Patients’ characteristics according to staging method

		All patients	CT staging	PET-CT staging	p-value
		N=99	N = 34	N = 65	
Gender	Male	69	22 (65%)	47 (72%)	0.434
	Female	30	12 (35%)	18 (28%)	
Age at diagnosis	Min-Max	21-78	25-74	21 - 78	0.043
	Median	49.5	46	53	
Histological subtype	Nodular sclerosis	65 (66%)	23 (68%)	42 (65%)	0.324
	Mixed cellularity	23 (23%)	9 (26%)	14 (22%)	
	Lymphocyte predominant	6 (6%)	0	6 (9%)	
	Unclassified	5 (5%)	2 (6%)	3 (5%)	
PS	0	76 (77%)	21 (62%)	45 (69%)	0.572
	1	22 (22%)	8 (24%)	14 (22%)	
	2	10 (10%)	4 (12%)	6 (9%)	
	3	1 (1%)	1 (3%)	0	
Ann Arbor stage	I	79 (20%)	3 (9%)	17 (26%)	0.041
	II	20 (80%)	31 (91%)	48 (74%)	
Bulky tumor (mass> 10 cm) (n=86)		10 (10%)	6 (17%)	4(6%)	0.206
Central disease		67 (68%)	28 (82%)	39 (60%)	0.024
Peripheral disease		73 (74%)	24 (71%)	49 (75%)	0.607
Spleen		9 (9%)	5 (14%)	4 (6%)	0.160
Hemoglobin g/dL (n=93)	Min-Max	8-16.6	8-15.6	8.9-16.6	0.015
	Median	13.2	12.8	13.6	
White blood cell G/L (n=87)	Min-Max	1.9-24.1	1.9-18.2	3.4-24.1	0.9
	Median	8.48	8.5	8.45	
Lymphocyte G/L (n=81)	Min-Max	0.27-7.9	0.5-3.1	0.27-7.9	0.08
	Median	1.54	1.38	1.7	
ESR mm (n=62)	Min-Max	2-125	3-125	2-120	0.022
	Median	42.5	58	27	
Period of diagnosis	< 2000	11 (11%)	11 (32%)	0	<0.001
	2000-2004	21 (21%)	19 (56%)	2 (3%)	
	2005-2009	44 (45%)	4 (12%)	40 (62%)	
	> 2010	23 (23%)	0	23 (35%)	

As compared to patients staged by CT-scan, PET-CT-staged patients were older (median age 53 years, p=0.043), more frequently had Ann Arbor stage I (26%, p=0.041), and central lymph nodes were less often involved (60%, p=0.024). In addition, they less frequently presented anemia and a high ESR.

### Treatment

Chemotherapy alone was performed in 54 patients (55%) while 45 patients (45%) had consolidation radiotherapy. As expected the median number of chemotherapy cycles was higher in the CT-staged group (6 cycles versus 4 cycles) (Table 2).

**Table 2:** Treatments and outcomes

		All patients	CT staging	PET-CT staging	p-value
		N=99	N = 34	N = 65	
Treatment	Combined chemoradiotherapy	45 (45%)	15 (44%)	30 (46.2%)	0.847
	**ABVD only**	54 (55%)	19 (56%)	35 (53.9%)	
	ABVD	99 (100%)	34 (100%)	65 (100%)	
Number of courses	Min-Max	1-8	3-8	1-8	0.083
	Median	5	6	4	
Response at the end of treatment, n(%)	Complete response	86 (87%)	28 (83%)	58 (89.2%)	0.514
	Partial response	9 (9%)	4 (12%)	5 (7.7%)	
	Progression	4 (4%)	2 (6%)	2 (3.1%)	
Vital status at 5 years	Deaths	11 (11%)	6 (18%)	5 (8%)	0.134
Relapse at 5 years, n(%)	Yes	20 (20%)	9 (26%)	11 (17%)	0.261
Causes of death	Hodgkin lymphoma	4 (36%)	1 (17%)	3 (60%)	0.259
	Second malignancies	2 (19%)	2 (33%)	0	
	Toxicity of chemotherapy	4 (36%)	2 (34%)	2 (40%)	
	Unknown	1 (9%)	1 (17%)	0	

The treatment modality did not differ between CT-staged and PET-CT-staged patients.

### Outcomes

With a median follow-up of 61.2 months (7-312 months), 24 patients progressed (n=4) or relapsed (n=20) within the five years following the diagnosis. The median time to treatment failure was 12.8 months. The 5-year PFS and OS rates were 72% (95%CI 61-80) and 86% (95%CI 76-92), respectively.

Eleven patients died (11%), four from HL, four from chemo-toxicity (infectious events), two from second malignancies and one patient from an unknown cause.

In the CT-staged group, the median follow-up of patients was 93.6 months (7-312 months). Ten relapses occurred (29%), including nine (26%) in the five years following the diagnosis of IDHL, after a median of 12.8 months (7-56 months). Seven patients died (20%), one from HL, two from the toxicity of the first-line chemotherapy (infectious events) and four from second malignancies. PFS and OS at 5 years reached 65% (95% CI 46-78) and 82% (95% CI 65-92), respectively.

**Figure 1:**
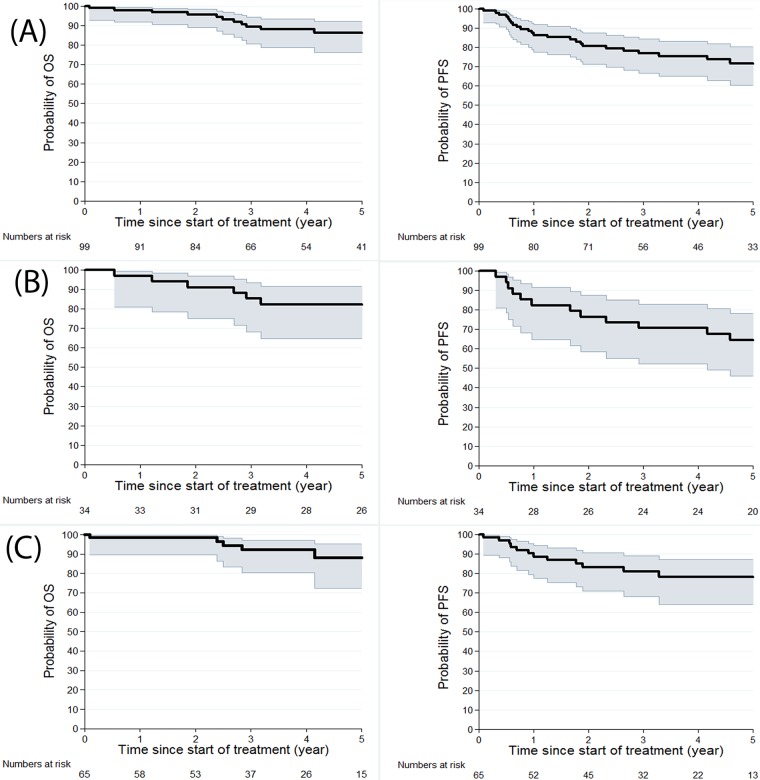
Kaplan Meier estimates for OS and PFS for 99 patients **(A)**, for CT-staged patients **(B)** and for PET-CT staged patients **(C)**.

In the PET-CT-staged group, the median follow-up was 46.8 months (range 1-125 months). Thirteen relapses (20%) occurred after a median of 21.6 months (4.5-104 months), among which 11 (17%) occurred in the five years after diagnosis. Five patients died (8%), three from HL and two from chemo-toxicity (infectious events). Five-year PFS and OS were 78% (95% CI 64-87) and 88.3% (95% CI 73-95), respectively (Figure 1).

Among the 20 patients who relapsed in the five years following diagnosis, 11 (55%) had advanced stage disease. In detail, in the subset of patients treated with chemotherapy alone, as mentioned in Table 2, nine relapses occurred with staging as follows: four with advanced stages, two without known staging and three with localized (I/II) relapse, one of which was in the infradiaphragmatic area. Concerning patients treated with chemoradiotherapy (CRT), one patient relapsed with an advanced stage, one with unknown status and two with a localized stage in the supradiaphragmatic area. In comparison, in the subset of PET-CT with CRT patients, three relapsed and of these patients, two had advanced-stage relapses and one had localized relapse in the same area.

**Figure 2:**
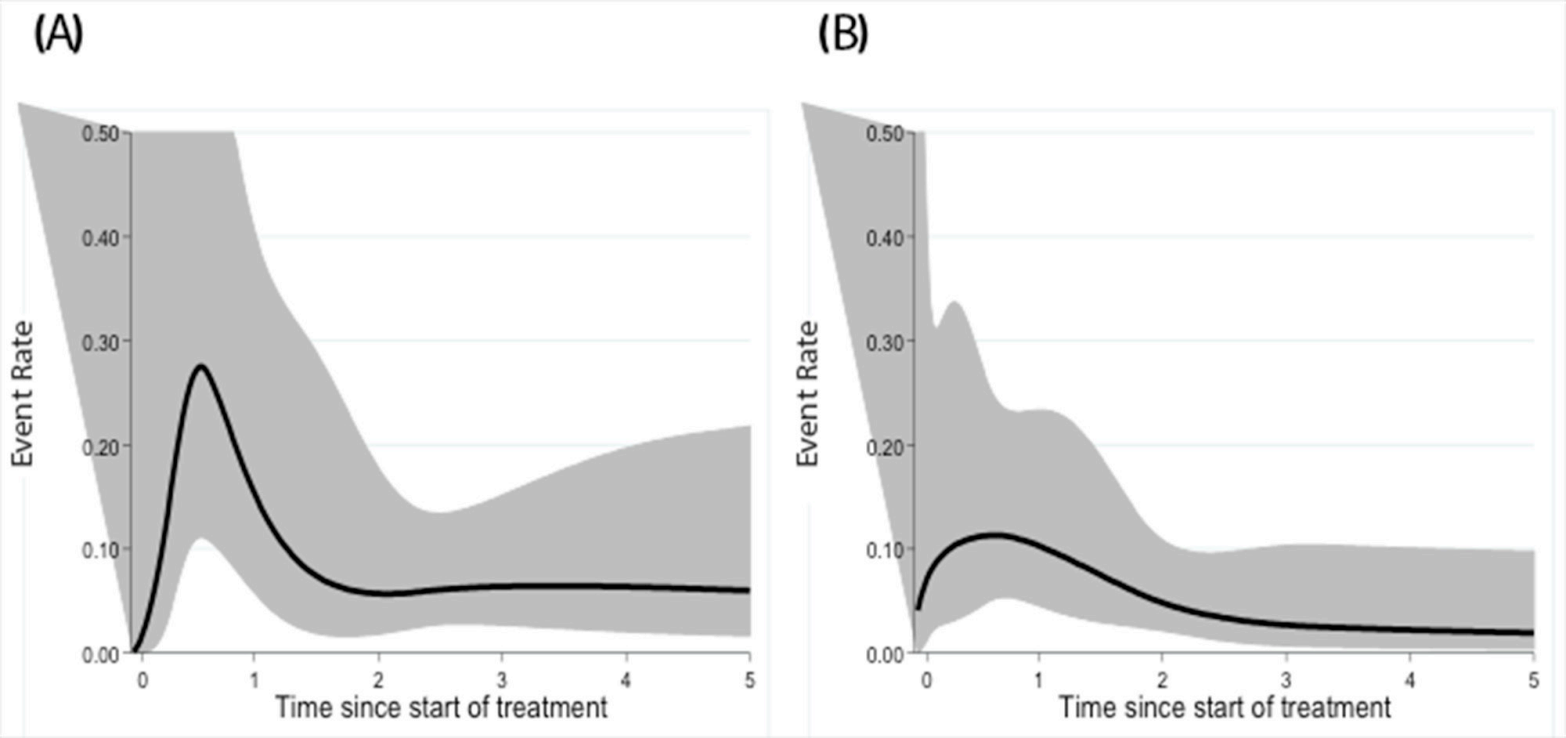
Dynamic profile of PFS for CT-staged **(A)** and PET-CT-staged patients **(B)**.

To compare timing of the onset of PFS events in CT-staged or PET-CT staged groups, the dynamics of the event hazard rate (pooling relapse and death) were analyzed using dynamic curves (Figure 2). In the CT-staged group, a peak of events occurred in the year following diagnosis. In addition, a plateau of events appeared after three years of follow-up for the CT-staged group. For PET-CT-staged patients, the frequency of events was maximal during the first year and decreased throughout the follow-up with no real early peak.

Overall survival was similar in both the CT-staged and PET-CT-staged groups.

### Prognostic factors

The full multivariate model included age at diagnosis, gender, performance status, hemoglobin, ESR, Ann Arbor stage, the presence of central adenopathy and/or peripheral disease and/or spleen involvement and treatment duration. Following the strategy described in the methods section, the parameters retained in the final model are shown in Table 3.

**Table 3:** Multivariate analysis of PFS for PET-CT staged patients

	HR (95% CI)	p value
**Age at diagnosis *(more than or equal to 54 y versus less than 54y)***	1.17 (0.23-6.05)	0.851
**Central adenopathy *(versus no central adenopathy)***	1.85 (0.07-49.3)	0.712
**Hemoglobin *(more than or equal to 13.5g/dL)***	0.85 (0.16-4.48)	0.834
**ESR *(more than or equal to 27mm versus less than 27mm)***	4.01 (0.15-106.06)	0.406
**Ann Arbor stage *(II versus I)***	0.07 (0.001-3.53)	0.185
**PS *(2-3 versus 0-1)***	11.39 (1.12-115.6)	0.040
**Treatment *(chemo-radiotherapy combined versus chemotherapy alone)***	0.08 (0.01-0.75)	0.027
**Duration of treatment *(>4 months versus < 4 months)***	0.73 (0.04-12.18)	1.05

In the CT-staged group, the final model did not retain any parameters significantly related to the risk of death or relapse.

**Figure 3:**
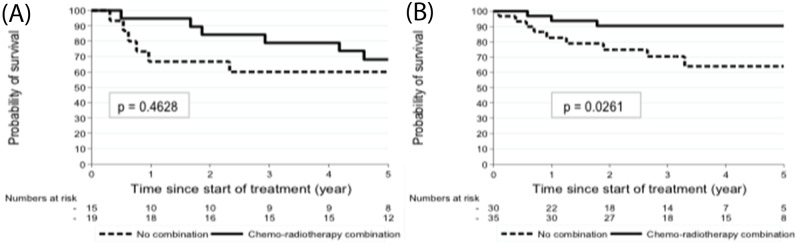
PFS of CT-staged **(A)** and PET-CT staged patients **(B)** according to the treatment.

In the PET-CT-staged group (Table 3), the PS score (2-3 versus 0-1) was inversely correlated with the event rate. In addition, the CRT strategy was a statistically significant factor to predict relapse, and this adjusted for other covariables (p=0.027). Figure 3 shows the comparison between CT-staged and PET-CT-staged patients according to the modality of treatment, which confirmed the significant impact for PET-CT staging on outcomes (p= 0.0261).

### Second malignancies

Ten second malignancies were reported (six in the CT-staged patients and four in the PET-CT-staged patients). These included 6 hematological malignancies (two acute myeloid leukemia, three lymphomas and one myelodysplasia) and four solid tumors (two melanomas, one sigmoid colon cancer and one cutaneous carcinoma). Six of the ten malignancies occurred among patients treated with CRT.

## DISCUSSION

To the best of our knowledge, this is the largest series of IDHL patients staged by PET-CT. The characteristics of IDHL patients staged by PET-CT were more favorable than those staged by CT alone.

As found in previous studies [2, 3, 5, 7, 9, 28], the baseline characteristics of IDHL patients are different from those of SDHL patients. The characteristics of patients in our cohort confirmed this specificity in the landscape of HL. IDHL patients were more frequently male and older. These differences regarding the age and the gender were reported in a large series in 2006 [5] in which the baseline characteristics of SDHL patients were compared with those of IDHL patients treated in the same center. However, in our study, these characteristics varied according to the staging method. PET-staged patients were older and the disease seemed to be less unfavorable, with less frequent stage II and less frequent central nodal involvement, lower levels of inflammatory parameters (ESR, hemoglobin) compared with CT-staged patients. In addition, PET-staged patients had a lower risk of relapse and a better outcome (PFS at 5-years at 78%) compared with previous reports, in which treatment failure ranged from 15% to 42% [5-7, 10, 12]. By contrast, in the SDHL series [29], the relapse rate ranged from only 5 to 10%, depending on the baseline risk factors. The explanations for this difference might be the higher median age of IDHL patients, since the relapse rate in IDHL series is as high as that in elderly HL series [30].

Outcomes in PET-staged IDHL patients were better than those in CT-staged patients. As PET is more sensitive than CT [14-18] in identifying nodal and extra-nodal involvement, disease extension in CT-staged patients could be underestimated, with under-staged advanced disease in some patients, as was the case in previously reported series in which PET was not used or only marginally used in disease staging. Therefore, PET-CT appears to be more accurate than CT in evaluating the disease at baseline and is necessary to ensure that the criteria of IDHL are fulfilled before defining the treatment strategy. The poor prognosis of IDHL reported in previous series could thus be partly related to the inappropriate treatment delivered in under-staged patients [31].

For the same reasons, the pattern of outcomes was different between PET-CT-staged and CT-staged patients. CT-staged patients had a higher risk of treatment failure due to underestimated advanced disease. This also suggests that the prognosis in PET-CT-staged IDHL may be close to that in SDHL, and argues for a similar management strategy for localized HL, whatever the anatomic location (supra or infra diaphragmatic) of the involved area. As regards the difference between the median follow-up in the PET-staged group and that in the CT-staged group, the explanation is that PET-CT has been available in France since 2004, which means that follow-up was shorter for these PET-CT-staged patients.

The present study suggests that combined chemo-radiotherapy provides better PFS than does chemotherapy alone in these patients treated with the same chemotherapy regimen (ABVD). Chemo-radiotherapy might therefore be more efficient to control the disease. None of the previous studies focusing on IDHL found this difference, perhaps because of the small number of patients included [3, 8-11, 32]. In SDHL, two large randomized phase III trials (the RAPID trial [33] and the H10 trial [34]) underlined the importance of radiotherapy consolidation to reduce the risk of relapse. Indeed, in the RAPID trial [33], which compared strategies with or without radiotherapy after three cycles of ABVD for patients reaching a negative PET, relapses were more frequent in the arm without radiotherapy (20 relapses, 9.5%) than in the arm with radiotherapy (8 relapses, 3.5%), with a median of follow-up of 5 years. In the H10 trial [34]., in the unfavorable subgroup, 74.8% had a negative PET after two cycles of ABVD, there were seven events (3%) in the radiotherapy arm compared with 16 events (6%) in the arm without radiotherapy, with a follow-up of 1.1 years. In addition a meta-analysis [35] concluded that a combined strategy was associated with a better PFS (HR=2.4, 95% CI 1.64-3.53). Therefore, in patients with localized HL, either IDHL or SDHL responding to chemotherapy, radiotherapy might reduce the risk of relapse. A longer follow-up is required in PET-staged patients to evaluate the risk of a second malignancy in patients who received or did not receive radiotherapy.

The present study, cannot address the question of the optimal field - IFRT (involved field radiotherapy) or INRT (involved node radiotherapy), which could minimize the risk of long-term side effects. In this series no additional morbidity was reported in patients who received radiotherapy.

As the outcome of IDHL patients in the PET era is similar to that in patients with unfavorable SDHL and as it is unrealistic to design a specific prospective study for the rare IDHL patients, our results suggest that prospective studies dedicated to localized HL could use similar treatment strategies in both IDHL and SDHL and enroll patients with early disease regardless the site of the disease. Then, IDHL patients could benefit from the same PET-guided treatment strategies as those developed for SDHL patients to better manage the balance between treatment efficacy and toxicity [34].

To conclude, this multicenter retrospective study of IDHL showed that the characteristics of PET-CT-staged IDHL patients were more favorable than those of CT-staged patients, thus suggesting that PET gives a more accurate diagnosis in patients with infradiaphragmatic disease and is thus necessary to determine the best treatment strategy. In this series, CRT provided better PFS as compared to chemotherapy alone. The inclusion of these patients in prospective studies mainly designed for SDHL could help define a standard of care for this small subset of HL patients.

## MATERIALS AND METHODS

### Patients

This retrospective study analyzed patients with stage I-II biopsy-proven infradiaphragmatic classical HL, according to the 2008 World Health Organization classification. [19]. Patients were treated with ABVD followed or not with radiotherapy. Patients with positive HIV serology or with bone marrow involvement or unavailable bone marrow status were excluded.

Staging was established according to the Cotswolds criteria [20]. A bulky mass was defined as a lymph node diameter > 10cm [20]. Baseline staging was performed according to CT-scan or PET-CT. Clinical and biological data were analyzed retrospectively. The type of therapy, the response to treatment and patients’ outcomes were evaluated.

As IDHL is a very uncommon entity, we recruited patients from centers which had an available database of HL patients so as to maximize the exhaustiveness of data in each center. Due to the poor prognosis reported in IDHL patients, most of these cases were referred to these university hospitals which have expertise in the treatment of HL patients. It is important to note that the proportion of patients in this subset was certainly lower during the PET-CT era than in reported series during the CT era, which confirms the need to focus on exhaustive data from reference centers to study these IDHL patients.

### Localization of involved nodes

As reported in previous IDHL series [2, 4, 5, 7, 10, 12, 21], nodal localizations were divided into central, including mesenteric and/or lumbar-aortic and/or iliac involvement, and peripheral, including inguinal and/or crural adenopathy.

### Treatment

Patients were treated according the recommendations of the Lymphoma Study Association (LYSA, formerly Groupe d’Etude des Lymphomes de l’Adulte) [22]. The planned treatment, had to be 4 to 6 cycles of ABVD, followed or not by radiotherapy according to the clinician’s decision. Patients treated with regimens other than ABVD or radiotherapy alone were not included in the study. Radiotherapy targeted involved fields, as recommended in several series [22, 23], and delivered 30 grays.

### Statistics

Patients’ characteristics were analyzed according to the baseline staging method, either PET-CT or CT. The two groups were compared using a Chi-square, or Fisher’s exact test as appropriate, and a Kruskall-Wallis for categorical and continuous variables, respectively, (p < 0.05 were considered significant). Median follow-up was calculated with the Kaplan-Meier reverse method [24].

The non-parametric Kaplan-Meier estimator was used to estimate all-cause survival (with 95% CI) and progression-free survival from the start of the first treatment by censoring the event at five years [25]. Overall survival (OS) was defined as the time between the date of the first treatment and the date of death due to any cause or date of the last follow-up. Progression-free survival (PFS) was the time between the date of the first treatment and the date of the first event (relapse or death), or the date of the last follow-up for patients with no events. A log-rank test was used to compare the survival of groups.

For each staging group, we used a parametric hazard model, the Royston and Parmar model, [26] which is more flexible than a classical Cox model. With this model, two parameters were studied: i) the dynamics of the occurrence of relapse and mortality over time from the beginning of treatment to five years; ii) the effects of covariates on relapse and mortality. The model building strategy was based on two steps. Firstly, as regards patients’ characteristics according to the staging group, models were adjusted for variables found to be significantly different between the two groups. Secondly, as proposed by Abrahamowicz et al. [27] we started with a full multivariate model adjusted for exposure time. To determine which model to retain, we used a backward elimination procedure. To analyze the prognostic value of quantitative parameters we dichotomized each of them according to the median value calculated in the PET-CT group.

Statistical analyses were performed using STATA.13 software.

## Abbreviations

IDHL: infradiaphragmatic Hodgkin Lymphoma
SDHL: supradiaphragmatic Hodgkin Lymphoma
HL: Hodgkin Lymphoma
PET-CT: Positron emission tomography-computer tomography
PFS: progression free survival
OS: overall survival
CRT: chemoradiotherapy
IFRT: involved field radiotherapy
INRT: involved node radiotherapy
ESR: elevated serum ratio
